# Geographical patterns of intraspecific genetic diversity reflect the adaptive potential of the coral *Pocillopora damicornis* species complex

**DOI:** 10.1371/journal.pone.0316380

**Published:** 2025-01-22

**Authors:** M. Carr, C. Kratochwill, T. Daly-Engel, T. Crombie, R. van Woesik

**Affiliations:** 1 Institute for Global Ecology, Florida Institute of Technology, Melbourne, Florida, United States of America; 2 Department of Ocean Engineering and Marine Sciences, Florida Institute of Technology, Melbourne, Florida, United States of America; 3 Department of Biomedical Engineering and Science, Florida Institute of Technology, Melbourne, Florida, United States of America; University of the Ryukyus, JAPAN

## Abstract

Marine heatwaves are increasing in intensity and frequency however, responses and survival of reef corals vary geographically. Geographical differences in thermal tolerance may be in part a consequence of intraspecific diversity, where high-diversity localities are more likely to support heat-tolerant alleles that promote survival through thermal stress. Here, we assessed geographical patterns of intraspecific genetic diversity in the ubiquitous coral *Pocillopora damicornis* species complex using 428 sequences of the Internal Transcribed Spacer 2 (ITS2) region across 44 sites in the Pacific and Indian Oceans. We focused on detecting genetic diversity hotspots, wherein some individuals are likely to possess gene variants that tolerate marine heatwaves. A deep-learning, multi-layer neural-network model showed that geographical location played a major role in intraspecific diversity, with mean sea-surface temperature and oceanic regions being the most influential predictor variables differentiating diversity. The highest estimate of intraspecific variation was recorded in French Polynesia and Southeast Asia. The corals on these reefs are more likely than corals elsewhere to harbor alleles with adaptive potential to survive climate change, so managers should prioritize high-diversity regions when forming conservation goals.

## Introduction

Coral reefs are one of the world’s most diverse ecosystems, supporting over 35% of all known marine species [1], and providing vital resources for millions of people globally [2]. However, coral reefs are degrading rapidly because of local pollution, diseases, and land-use changes [3]. More concerning are the global emissions of greenhouse gases that increase the intensity, frequency, and extent of marine heatwaves [4]. Marine heatwaves cause widespread coral bleaching and mortality [5–7]. Yet the response to thermal stress varies within individual coral colonies [8], among species [9], among habitats [10], and across regions and oceans [7, 11]. While several factors may explain such differences in thermal tolerance, here we are interested in examining geographical differences in the intraspecific genetic diversity of *Pocillopora damicornis* species complex in the Indian and Pacific Oceans. We investigate where corals may support high-standing intraspecific genetic diversity, which potentially increases the likelihood that some individuals may harbor variants that promote survival under natural and anthropogenic disturbances.

Differences in intraspecific genetic diversity may arise from the same evolutionary mechanisms that shape interspecific variations, such as geographical conditions, isolation-by-distance, phylogenetic ancestry, and historical bottlenecks [12]. Many natural evolutionary processes can change the diversity of a population at a given locality, including migration, genetic drift, and selection pressure. While genetic diversity is thought to be maintained through balancing selection, extreme ocean warming may cause strong directional selection, resulting in rapid adaptation through novel heat-tolerant alleles that promote survival [13]. These processes can lead to beneficial mutations becoming fixed, especially in small-isolated populations that experience strong genetic drift. As a consequence, locally-adapted alleles in areas that historically experience thermal stress [14] may be promoted at the expense of overall diversity [15], such that diversity itself is not always a direct predictor of survival.

Yet, intraspecific genetic variation is known to provide the raw material for adaptation, and highly-diverse populations of a given species are more resistant to extinction because they harbor variants that prove adaptive to changes in unpredictable environments, including climate-driven warming [13, 16, 17]. For example, high intraspecific variability helps fish populations adapt to warmer water [18], and seagrass populations with high intraspecific diversity have higher survival rates through physical [19] and thermal [20] disturbances. Similarly, increasing intraspecific genetic diversity has been shown to increase coral survival at high temperatures [21]. High genetic diversity in such reef-building organisms as corals, whose growth and structure shape the entire ecosystem [22], can also enhance habitat complexity. This has led investigators to hypothesize that such diverse “hotspots” may tolerate anthropogenic ocean warming and serve as climate refugia [23]. Thus, while it is not known whether directional or balancing selection is acting at a given moment, high genetic diversity is known to be associated with stress resistance and adaptive potential, and the loss of such diversity is likely to decrease survival in the face of modern climate change [24].

Here we examine geographical differences in the standing genetic diversity of the coral species *Pocillopora damicornis* species complex (herein after *Pocillopora damicornis*), which we chose for the following five reasons. Firstly, *P*. *damicornis* can reproduce sexually and asexually, depending on geographical location [25, 26]. Secondly, *P*. *damicornis* has been shown to hybridize and form a species complex [27], a group of closely related but morphologically- and genetically-distinct subspecies, all potentially inhabiting different niches and responding differently to disturbances [28]. Thirdly, considerable genetic data are publicly available for *P*. *damicornis* in online repositories. Fourthly, *P*. *damicornis* is widely distributed throughout the Indian and Pacific Oceans. Fifthly, other researchers have focused on regional trends in population structure for *P*. *damicornis* [29, 30], but not on across-ocean comparisons.

While the ubiquitous nature of *P*. *damicornis* is well known across the Indian and Pacific Oceans, the geographical pattern of intraspecific genetic diversity of *P*. *damicornis* is not. The Coral Triangle region encompassing the Philippines, Indonesia, Malaysia, Papua New Guinea, Timor-Leste, and the Solomon Islands supports the highest coral species diversity [31] as the region is known to be both a source and sink of marine evolutionary variation [32, 33]. Because of this trend, the geographical pattern of intraspecific genetic diversity may follow the trend of elevated interspecific diversity, and thus the intraspecific genetic diversity in *P*. *damicornis* may be highest in the Coral Triangle. However, there are few sequences available for *P*. *damicornis* in the Coral Triangle, and therefore we hypothesize that background ocean temperatures are a reasonable predictor of intraspecific diversity. Testing this hypothesis is pertinent as we seek to identify reef locations that may potentially act as climate-change refugia with high adaptive potential [34], providing valuable information for conservation [35, 36].

Any method used to examine genetic diversity must be able to differentiate among individuals from different populations [37]. Ribosomal DNA (rDNA) has been shown to evolve at a faster rate than other loci in acroporid corals [38] and is therefore suited to provide high-resolution estimates of population-level variation across geographical scales [39]. The rDNA gene cluster includes the 18S, 5.8S, and 28S rRNA genes, plus Internal Transcribed Spacers (ITS) 1 and 2. We used the selectively-neutral ITS region because of its appropriateness for the large geographical scale of the research, across the Indian and Pacific Oceans, and because its ubiquity as a tool meant that many sequences were publicly available for this analysis. Specifically, we examined the ITS2 region from *P*. *damicornis* across the Indian and Pacific Oceans to (1) determine geographical patterns of intraspecific nucleotide diversity, and (2) examine whether the intraspecific diversity was a function of any of eight predictor variables related to abiotic conditions and geography. As climate change continues to impact ocean habitats globally, understanding spatial patterns of intraspecific genetic diversity may provide insight into the survival mechanisms of some of the world’s most vulnerable ecosystems.

## Methods

### Sampling strategy

**Fig 1:**
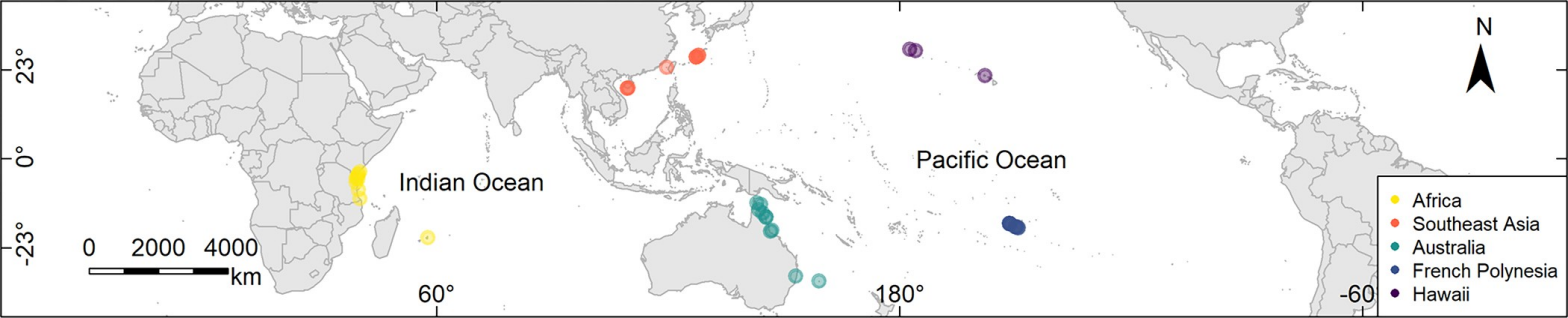
Geographical distribution of *Pocillopora damicornis* samples. The spatial distribution of the 44 *Pocillopora damicornis* sampling sites used in this study in the Pacific and Indian Oceans from 2005 to 2020. The colored points depicted in the key represent five ocean regions. The points representing sites with few coral samples are less opaque.

Ribosomal DNA (rDNA) sequence data including the nuclear Internal Transcribed Spacer 2 (ITS2) of *Pocillopora damicornis* were collected from GenBank [40]. Sequences were identified by searching the key terms “*Pocillopora damicornis* ITS2” or “*Pocillopora damicornis* internal transcribed spacer two”. Sequences that contained ITS2 region, and portions of neighboring gene regions were used in this study. All sequences with > 3% sequence divergence from known *P*. *damicornis* accessions were filtered from our dataset to avoid potentially mislabeled samples that likely belonged to other established species. The resulting dataset includes 428 DNA sequences that were added to Genbank from 2005 to 2020 (S1 File). Wherever possible, the geographical location of each sampling site was taken from the latitude and longitude coordinates provided in the source publication. In the absence of coordinates, the latitude and longitude were determined using Google Earth [41] based on locations or maps provided in the associated publication. This strategy resulted in 44 unique sampling sites that contained a possible range of 1 to 32 individual DNA sequences. The final set of 428 sequences from 44 sites (Fig 1) was uploaded into Geneious Prime 2023 [42] using the *NCBI Nucleotide* plugin tool.

The raw downloaded sequences were cleaned using the following seven methods in which the sequence ends were: (i) trimmed, (ii) trimmed and indels removed, (iii) trimmed and cleaned, (iv) cleaned and one gap left for indels, (v) trimmed, cleaned, and all indels removed, (vi) trimmed, cleaned, indels removed, and transversions cleaned, and (vii) trimmed, cleaned, indels removed, and point mutation cleaned (all data are available at https://github.com/InstituteForGlobalEcology/Pocillopora). We saw similarities in genetic diversity across sites and regions before and after cleaning (S1 and S2 Figs in S1 File), therefore we used the data with only the sequence ends trimmed, without any further cleaning or alterations, for further analyses in R [43]. The deep-learning neural-network results for the trimmed and the cleaned data were virtually identical (S3 Fig in S1 File).

### Data analysis

For each sampling site with three or more samples, genetic diversity, as measured by nucleotide diversity, was calculated as the average number of pairwise sequence differences per base pair using the *pegas* package [44]. Genetic diversity was used as the response variable in the analysis. The eight predictor variables used in the analysis were: (i) ocean, (ii) ocean region, (iii) ecoregion, (iv) latitude, (v) longitude, (vi) reef density (i.e., the number of reef centroids within 500 km of a given study site), (vii) mean sea-surface temperature (⁰C) from 2002 to 2023, and (viii) the number of samples per study site. More information on each variable is provided below.

The five ocean regions were: Africa, Southeast Asia, Australia, French Polynesia, and Hawaii; and where the ten ecoregions were: (i) the central and northern Great Barrier Reef, Australia (ii) French Polynesia, (iii) east Hawaii, (iv) Kenya and Tanzania, Africa, (v) Lord Howe Island, Australia, (vi) Mauritius, Africa, (vii) Ryukyu Islands, Okinawa, Japan, (viii) Solitary Islands, French Polynesia, (ix) the South China Sea, and (x) Torres Strait and the far northern Great Barrier Reef, Australia. Reef density and mean sea-surface temperatures were examined to determine whether there were relationships between the eight predictor variables and the genetic diversity of *P*. *damicornis*. The number of samples per study site was used in the model to determine whether sampling effort played a role in intraspecific genetic diversity. All continuous variables were examined for autocorrelation (S4 Fig in S1 File). The first five predictor variables were used in the model to examine the geographical scale of variability in genetic diversity.

Using a global reef shapefile, we generated a metric to measure reef density [45]. The centroids of each reef polygon were identified using the *raster* package [46], and the density of reef centroids within 500 km (250 km radius) of each site was calculated. Mean sea-surface temperature (⁰C) acquired from NASA Earth Observation from 2002 to 2023 (https://neo.gsfc.nasa.gov) was examined to determine the potential role that ocean temperature plays in the intraspecific genetic diversity of *P*. *damicornis*.

To characterize the relationship between the intraspecific genetic diversity of *P*. *damicornis* and environmental variables and geographical features, we used the Artificial Intelligence deep-learning platform H2O.ai, coded through R using *h2o* [47, 48]. Using this platform, we constructed a supervised deep-learning, multi-layer feedforward neural-network model. The data were partitioned into three folds, using 70% to train, 15% to validate, and 15% to test the model. We evaluated a suite of models using a random grid algorithm to optimize hidden layers and L1 regularization. The most optimal model, with the lowest deviance, had 32 hidden layers, an L1 regularization value of 0.00042, 10 epochs, three stopping rounds, a 0.005 stopping tolerance, and cross-validation.

Partial dependency plots were used to examine the relationships between genetic diversity and the eight predictor variables mentioned above. Following the implementation of the deep-learning model, we examined variable importance using the R package *imp* [49], which reshuffles each predictive variable in the model and measures the drop in model performance [50]. Loss in model performance was determined using the mean-absolute error, representing the average-absolute difference between actual and predicted values.

Finally, to examine the role of geographical species origin in our analysis, we built a phylogeny using the *tree builder* plugin for Geneious to construct a neighbor-joining maximum likelihood tree with bootstrap resampling. The software *jModelTest* [51] was used to derive the most likely mutational model for our data based on Aikake Information Criteria, and ITS2 sequences from three congeneric species (*Pocillopora meandrina*, *P*. *ligulata*, and *P*. *molokensis*) were downloaded from Genbank to serve as taxonomically-diverse outgroups (accession numbers in S2 File). We discarded all samples with >3% sequence divergence and verified that all sequences grouped in our phylogenetic tree, and well away from the outgroup. All data and R code used in the analyses are available at https://github.com/InstituteForGlobalEcology/Pocillopora.

## Results

**Fig 2:**
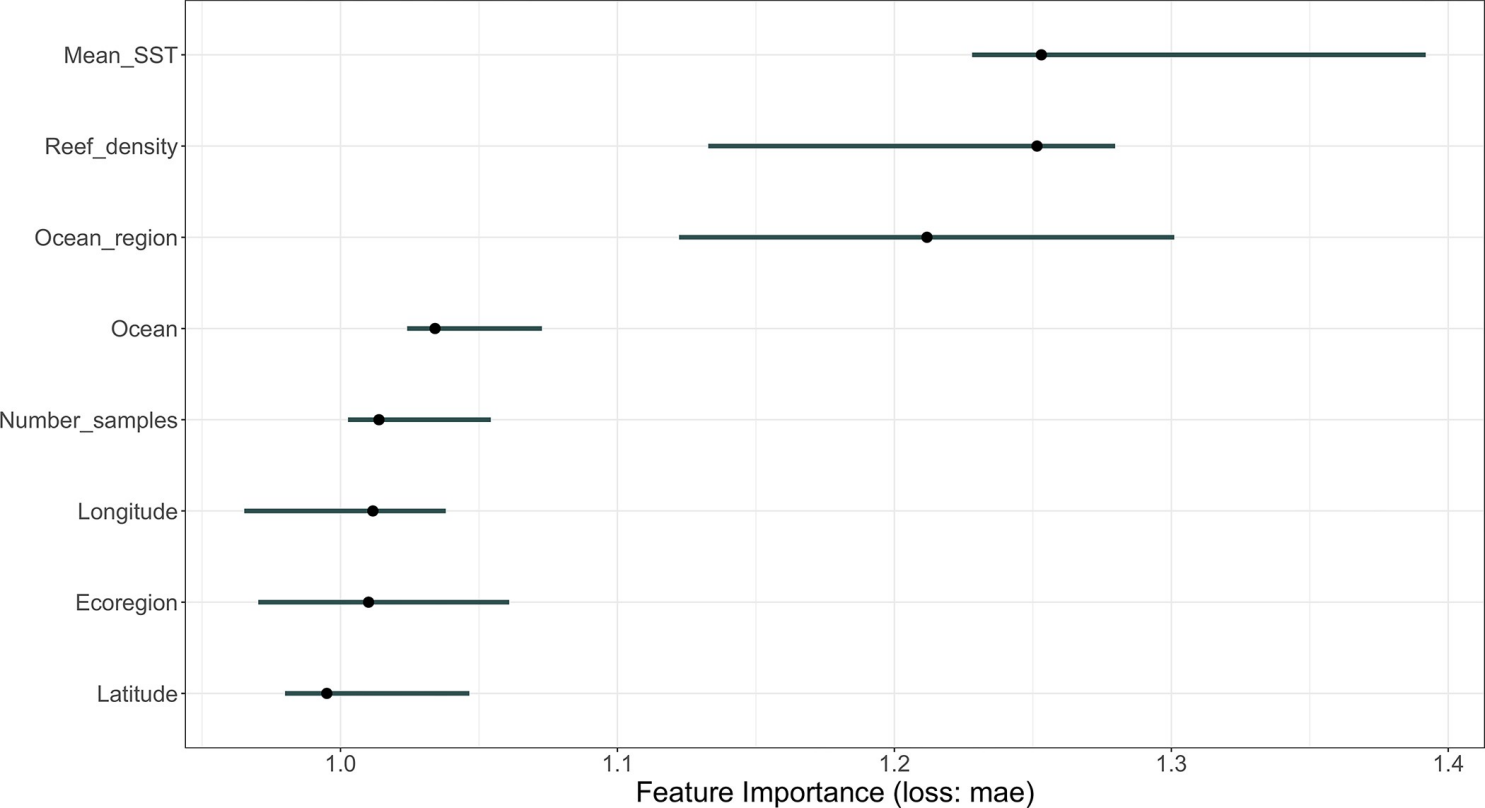
Variable importance plot. Variable importance plot using results from the deep-learning neural network model showing the relative influence of eight predictor variables on the genetic diversity of *Pocillopora damicornis* across 44 sites in five ocean regions, in the Pacific and Indian Oceans, from 2005 to 2020. The points are medians, and the whiskers are 5% and 95% quantiles. The eight predictor variables were: (i) ocean, (ii) ocean region, (iii) ecoregion, (iv) latitude, (v) longitude, (vi) reef density (i.e., the number of reef centroids within 500 km of a given study site), (vii) mean sea-surface temperature (⁰C), and (viii) the number of samples per study site. The five ocean regions (as depicted in Fig 1) were: Africa, Southeast Asia, Australia, French Polynesia, and Hawaii.

**Fig 3:**
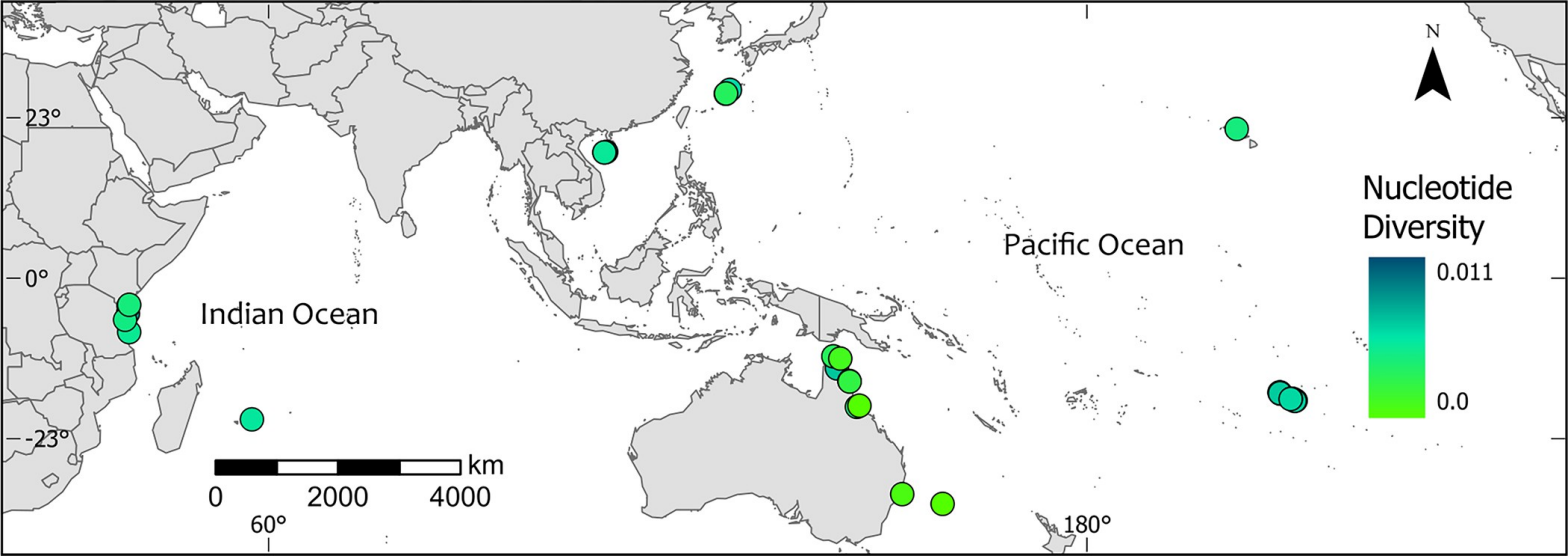
Nucleotide diversity. Map of nucleotide diversity of the coral *Pocillopora damicornis* among 34 sites (each with ≥ 3 samples) in five ocean regions in the Pacific and Indian Oceans (from 2005 to 2020). Dark-green sites have high nucleotide diversity and light-green sites have low nucleotide diversity. The five ocean regions (as depicted in Fig 1) were Africa, Southeast Asia, Australia, French Polynesia, and Hawaii.

The deep-learning neural network used to estimate the relative importance of predictive variables on genetic diversity showed that mean sea-surface temperature and geographical location (i.e., ocean region) had the greatest influence on the genetic-diversity model (Fig 2). The highest intraspecific genetic diversity was apparent in French Polynesia, in the South Pacific Ocean, and Southeast Asia, in the Western Pacific Ocean, and the lowest was in Hawaii, the central and southern Great Barrier Reef, the Solitary Islands, and Lord Howe Island, Australia (Fig 3). Intraspecific diversity was moderately high in Kenya, Tanzania, and Mauritius in the Indian Ocean, and in the Torres Strait and the far northern Great Barrier Reef, in the Pacific Ocean (Fig 3). The deep-learning model showed that reef density (i.e., the density of reef centroids within 500 km) had a relationship with genetic diversity (Fig 2). The oceans, the number of samples, longitude, latitude, and ecoregion were not particularly influential on the genetic diversity of *Pocillopora damicornis* (Fig 2).

**Fig 4:**
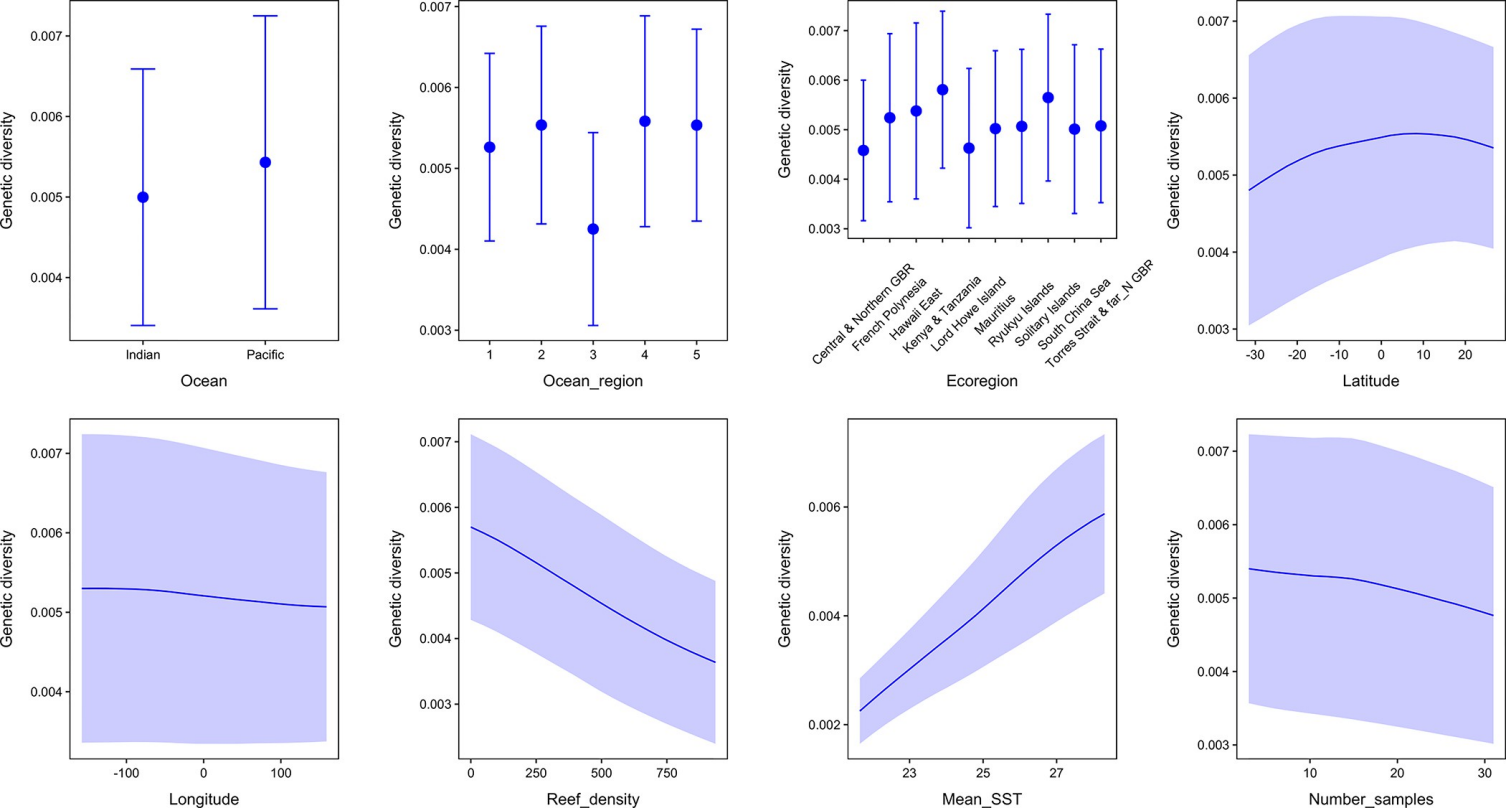
Partial dependency plots. Partial dependency plots showing the relationships of eight predictor variables to genetic diversity in *Pocillopora damicornis* across 44 sites in five ocean regions in the Pacific and Indian Oceans, from 2005 to 2020, as determined by the deep-learning neural network. The response variable was the nucleotide diversity of the Internal Transcribed Spacer 2 (ITS2) and flanking regions at each of the sites. The points are means and the whiskers and purple shading are standard deviations. The eight predictor variables were (i) ocean, (ii) ocean region, (iii) ecoregion, (iv) latitude, (v) longitude, (vi) reef density (i.e., the number of reef centroids within 500 km of a given study site), (vii) mean sea-surface temperature (°C), and (viii) the number of samples per study site. The five ocean regions (as depicted in Fig 1) were: Africa, Southeast Asia, Australia, French Polynesia, and Hawaii. The ten ecoregions were (i) the central and northern Great Barrier Reef, Australia (ii) French Polynesia, (iii) east Hawaii, (iv) Kenya and Tanzania, Africa, (v) Lord Howe Island, Australia, (vi) Mauritius, Africa, (vii) Ryukyu Islands, Okinawa, Japan, (viii) Solitary Islands, Australia, (ix) South China Sea, and (x) Torres Strait and the far northern Great Barrier Reef, Australia.

The partial dependency plots showed a strong positive relationship between the 21-year mean sea-surface temperature and intraspecific genetic diversity of *Pocillopora damicornis*, with the highest diversity found at reefs with mean temperatures between 26°C and 28°C (Fig 4). Reef density had a negative relationship with genetic diversity mainly because corals in French Polynesia had high genetic diversity and relatively low reef density. The overall intraspecific genetic diversity of *P*. *damicornis* was higher in the Pacific than in the Indian Ocean, although the difference was slight (Fig 4). Diversity varied considerably across oceanic regions, with the East Australian sites supporting the lowest diversity. Interestingly, the number of samples at each site, longitude, and latitude had little influence on overall genetic diversity (Fig 4).

The *jModelTest* inferred the most likely mutational model for our data was Tamura-Nei with no gamma correction, which was input to the Geneious tree builder before analysis. The resulting phylogenetic tree showed relatively low overall discriminatory variation but enough to reflect the subtle geographical differences in our diversity analyses (S2 File). Specifically, the basal clade contained samples primarily from French Polynesia, with some representation from Hawaii, China, Australia, and East Africa. The more derived clades show separate incursions into the broader region, including novel lineages in China, southern Japan, French Polynesia, Australia, Hawaii, and especially east Africa.

## Discussion

The deep-learning model showed a strong influence of geographical location on the intraspecific nucleotide diversity of *Pocillopora damicornis*. Genetic diversity was particularly dependent on oceanic regions and not simply a consequence of latitude or longitude. The highest intraspecific diversity of *P*. *damicornis* was found in French Polynesia and Southeast Asia, and the lowest was found in Hawaii and the Great Barrier Reef, Australia. High intraspecific diversity in a given locality is thought to help preserve populations that experience stressful environmental events [14]. Because selection acts upon traits that already exist within a population, high genetic variation increases the likelihood that some individuals will survive the stressors of the environment [52]. In *Acropora palmata*, for example, Parkinson et al. [53] found that the stress response to cold water differed among individuals with the same symbionts, but with different coral genotypes. Gillette [54] also found that the thermal tolerance of corals was more dependent on the coral genotype than the endosymbionts and the microbiome, suggesting the possibility of local adaptation.

We found a negative relationship between reef density and genetic diversity, likely driven in part by the low reef density and high genetic diversity in French Polynesia. Though consistent with the theoretical expectation that isolated reefs experience genetic drift more strongly than connected ones, this is surprising because it is known that high-density reefs can be more resilient to climate change [55], and the absence of neighboring reefs increases climate vulnerability [56]. Adaptation to environmental stress is assumed to occur in large metapopulations through a mix of dispersal and isolation, which could facilitate an adaptive response to selection pressure [13, 17]. High gene flow among dense reefs may cause alleles to homogenize before local adaptation can develop, whereas high genetic drift among isolated reefs can cause alleles to be culled even if they provide a selective advantage. However, moderate gene flow among semi-isolated subpopulations can promote the evolution of locally-adapted alleles, while still allowing those alleles to spread to neighboring populations, increasing diversity in the region as a whole [12].

Moderate gene flow and relative isolation from other reefs in the Pacific Ocean may be responsible for the high diversity we observed in French Polynesia, which extends from 7 to 17°S and 134 to 155°W, supporting 130 islands, 78 atolls, and 6,340 km^2^ of coral reefs. This region is a hotspot for marine diversity, likely because of a combination of geographical circumstances, historical vicissitudes, and the relative isolation of French Polynesia from other Pacific reefs. Modern oceanographic observations in French Polynesia show a weak and shallow westward flow of the South Equatorial Current that is frequently interrupted by eastward countercurrents, particularly during El Niño Southern Oscillation events [57]. These events may cause connectivity to fluctuate, causing shifts in the rate of genetic drift and local adaptation among subpopulations. High intraspecific diversity in *P*. *damicornis* in French Polynesia and Southeast Asia is likely to enhance the chances of those populations surviving future thermal stress events, and potentially benefit neighboring ecoregion populations [14, 20].

*Pocillopora* is thought to have diverged from *Stylophora* some 56 million years ago at the beginning of the Eocene [58]. The ancestry of *P*. *damicornis* diverged from *P*. *eydouxi* (now called *P*. *grandis* according to the World Register of Marine Species) around 7.5 million years ago [59] (S5 Fig in S1 File), and the species is thought to have originated ~1 million years ago [58]. The present study found the Indo-West Pacific to be a potential site of origin for *P*. *damicornis* (S2 File), though examination with additional loci is needed to resolve this question. This is because of the relatively low variation observed at ITS2, which was chosen for its ubiquity among genetic studies on coral rather than its ability to discriminate between sites. Nonetheless, the fact that the basal clade in our tree contains genotypes from most sites included in this study suggests that geographical origin does not play a role in shaping patterns of diversity.

Many studies have investigated diversity in the Coral Triangle, which harbors the highest species-level variation of many reef-associated organisms, including scleractinian corals [31]. These studies have hypothesized that the area: (i) is the center of origin of many tropical and subtropical species [60], (ii) supports more derived genotypes than in the central Pacific Ocean [61], and (iii) is the center of overlap for some species [62] but the center of accumulation for others [63, 64]. In a meta-analysis in the Pacific Ocean, Chaudhary and Kastner [65] found species richness peaked at latitudes 15° north and south of the Equator. A similar trend was observed for foraminiferal diversity [66]. Our study found little influence of latitude and longitude on the genetic diversity of *P*. *damicornis*, although there was a diversity peak at 15° south of the equator, but no second peak in the northern latitudes.

Data scarcity imposed several limitations on this study. While we gathered all the ITS2 sequences available in GenBank, many regions across the Indian and Pacific Oceans were not represented. We also examined the *P*. *damicornis* species complex as a whole, realizing that the complexities of this species and its response to climate change may differ within the species complex [67]. Moreover, we used the ITS2 gene for this study, but mitochondrial genes [68] and rapidly-evolving nuclear markers such as microsatellites [69] have also been used to study diversity patterns. Since such loci mutate at different rates and under different evolutionary models, they reflect different temporal scales of evolutionary history, so different methods could obtain slightly different results. Further studies examining other genetic markers in additional species across the Indo-Pacific will provide greater insight into the geographical variation of intraspecific genetic diversity in corals.

Extreme thermal stressors like marine heatwaves exert strong directional selection on coral reefs, which can fix beneficial heat-tolerant mutations, particularly in small, isolated populations that experience genetic drift. Therefore, diversity may only be a good predictor of survival through stress events in some cases, as low diversity within a particular site or region may reflect a history of local heat adaptation. In that scenario, thermally tolerant alleles are promoted at the expense of overall diversity at frequently stressed sites [70]. Though we did not address this particular hypothesis, we emphasize that conservation decisions based solely on genetic diversity would erroneously ignore those heat-tolerant sites. With further information on intraspecific genetic diversity, conservation plans can target locations that have high potential survivability. Restoration and outplanting efforts can be more effective when armed with information on the intraspecific diversity of stock populations, by prioritizing the outplanting of heat-tolerant variants and reducing the potential for genetic incompatibility among propagules.

In conclusion, we highlight patterns of intraspecific diversity of *Pocillopora damicornis* in the Indian and Pacific Oceans, providing insight into geographical differences in the potential of coral populations to adapt to climate change. We found that the reefs in French Polynesia in the South Pacific Ocean and reefs in Southeast Asia in the Western Pacific Ocean support high intraspecific diversity, with a strong likelihood of harboring individuals capable of surviving future thermal stress events. Geographical vicissitudes and high mean temperatures led to higher intraspecific diversity in the past, however anomalously high temperatures associated with climate change will likely reshape patterns of genetic diversity. Ultimately, genome-wide analyses are required to identify adaptive alleles and pinpoint reef populations that harbor genetic variants with the potential to withstand climate-induced stresses. However, until specific adaptive alleles are found, the genetically diverse corals on these reefs should be given the highest conservation status, as they are most likely to harbor adaptive alleles.

## Supporting information

S1 File Supplementary methods and figures. (PDF)

S2 File Phylogenetic tree of *Pocillopora damicornis*. (PDF)

## References

[1] Reaka-Kudla ML. Biodiversity of Caribbean coral reefs. In: Miloslavich P, Klein E, editors. Caribbean Marine Biodiversity: The Known and the Unknown. 2005; 259–276.

[2] Costanza R, de Groot R, Sutton P, van der Ploeg S, Anderson SJ, Kubiszewski I, et al. Changes in the global value of ecosystem services. Global Environmental Change: Human and Policy Dimensions. 2014; 26: 152–158.

[3] Nyström M, Folke C, Moberg F. Coral reef disturbance and resilience in a human-dominated environment. Trends in Ecology & Evolution. 2000; 15: 413–417. 10998519 10.1016/s0169-5347(00)01948-0

[4] Heron SF, Maynard JA, van Hooidonk R, Eakin CM. Warming trends and bleaching stress of the world’s coral reefs 1985–2012. Scientific Reports. 2016; 6: 38402. doi: 10.1038/srep38402 27922080 PMC5138844

[5] Glynn PW. Coral reef bleaching: ecological perspectives. Coral reefs. 1993; 12: 1–17.

[6] Hoegh-Guldberg O. Climate change, coral bleaching and the future of the world’s coral reefs. Marine and Freshwater Research. 1999; 50: 839–866.

[7] Sully S, Burkepile DE, Donovan MK, Hodgson G, van Woesik R. A global analysis of coral bleaching over the past two decades. Nature Communications. 2019; 10: 1264. doi: 10.1038/s41467-019-09238-2 30894534 PMC6427037

[8] Brown B, Dunne R, Goodson M, Douglas A. Experience shapes the susceptibility of a reef coral to bleaching. Coral Reefs. 2002; 21: 119–126.

[9] Loya Y, Sakai K, Yamazato K, Nakano Y, Sambali H, van Woesik R. Coral bleaching: the winners and the losers. Ecology Letters. 2001; 4: 122–131.

[10] van Woesik R, Sakai K, Ganase A, Loya Y. Revisiting the winners and the losers a decade after coral bleaching. Marine Ecology Progress Series. 2011; 434: 67–76.

[11] Shelsinger T, van Woesik R. Oceanic differences in coral-bleaching responses to marine heatwaves. Science of the Total Environment. 2023; 871: 162113. doi: 10.1016/j.scitotenv.2023.162113 36773903

[12] Avise JC. Phylogeography: The History and Formation of Species. Harvard University Press; 2000, pp 447.

[13] Barrick JE, Lenski RE. Genome dynamics during experimental evolution. Nature Reviews. Genetics. 2013; 14: 827–839. doi: 10.1038/nrg3564 24166031 PMC4239992

[14] Matz MV, Treml EA, Aglyamova GV, Bay LK. Potential and limits for rapid genetic adaptation to warming in a Great Barrier Reef coral. PLoS Genetics. 2018; 14: e1007220. doi: 10.1371/journal.pgen.1007220 29672529 PMC5908067

[15] Ehlers A, Worm B, Reusch TB. Importance of genetic diversity in eelgrass Zostera marina for its resilience to global warming. Marine Ecology Progress Series. 2008; 355: 1–7.

[16] Humanes A, Lachs L, Beauchamp EA, Bythell JC, Edwards AJ, Golbuu Y, et al. Within-population variability in coral heat tolerance indicates climate adaptation potential. Proc. R. Soc. B. 2022; 289: 1–11. doi: 10.1098/rspb.2022.0872 36043280 PMC9428547

[17] Pinsky ML, Clark RD, Bos JT. Coral reef population genomics in an age of global change. Annual Review of Genetics. 2023; 57: 87–115. doi: 10.1146/annurev-genet-022123-102748 37384733

[18] McKenzie DJ, Zhang Y, Eliason EJ, Schulte PM., Claireaux G, Blasco FR, et al. Intraspecific variation in tolerance of warming in fishes. Journal of Fish Biology. 2021; 98: 1536–1555. doi: 10.1111/jfb.14620 33216368

[19] Hughes AR, Stachowicz JJ. Genetic diversity enhances the resistance of a seagrass ecosystem to disturbance. Proceedings of the National Academy of Sciences of the United States of America. 2004; 101: 8998–9002. doi: 10.1073/pnas.0402642101 15184681 PMC428461

[20] Reusch TBH, Ehlers A, Hämmerli A, Worm B. Ecosystem recovery after climatic extremes enhanced by genotypic diversity. Proceedings of the National Academy of Sciences of the United States of America. 2005; 102: 2826–2831. doi: 10.1073/pnas.0500008102 15710890 PMC549506

[21] Huffmyer AS, Bean NK, Majerová E, Harris CI, Drury C. Variable intraspecific genetic diversity effects impact thermal tolerance in a reef-building coral. Coral Reefs. 2023; 42: 119–129.

[22] Wild C, Hoegh-Guldberg O, Naumann MS, Colombo-Pallotta MF, Ateweberhan M, Fitt WK, et al. Climate change impedes scleractinian corals as primary reef ecosystem engineers. Marine and Freshwater Research. 2011; 62: 205–215.

[23] van Woesik R, Houk P, Isechal AL, Idechong JW, Victor S, Golbuu Y. Climate-change microrefugia: nearshore reefs bleach less than outer reefs during a 2010 regional thermal stress event in Palau. Ecology and Evolution. 2012; 2: 2474–2484. 23145333 10.1002/ece3.363 PMC3492774

[24] Pauls SU, Nowak C, Bálint M, and Pfenninger M. The impact of global climate change on genetic diversity within populations and species. Molecular Ecology. 2013; 22(4), 925–946. doi: 10.1111/mec.12152 23279006

[25] Baird AH, Guest JR, Edwards AJ, Bauman AG, Bouwmeester J, Mera H, et al. An Indo-Pacific coral spawning database. Scientific Data. 2021; 8: 1–9. 33514754 10.1038/s41597-020-00793-8 PMC7846567

[26] Stoddart JA. Asexual production of planulae in the coral *Pocillopora damicornis*. Marine Biology. 1983; 76: 279–284.

[27] Schmidt-Roach S, Lundgren P, Miller KJ, Gerlach G, Noreen AME, Andreakis N. Assessing hidden species diversity in the coral *Pocillopora damicornis* from Eastern Australia. Coral Reefs. 2013; 32: 161–172.

[28] Johnston EC, Forsman ZH, Flot JF, Schmidt-Roach S, Pinzon JH, Knapp ISS, et al. A genomic glance through the fog of plasticity and diversification in *Pocillopora*. Scientific Reports 2017; 7, 5991. doi: 10.1038/s41598-017-06085-3 28729652 PMC5519588

[29] Chávez-Romo HE, Correa-Sandoval F, Paz-García DA, Reyes-Bonilla H, López-Pérez RA, Medina-Rosas P, et al. Genetic structure of the scleractinian coral, *Pocillopora damicornis*, from the Mexican Pacific. Proceedings of the 11th International Coral Reef Symposium; 2008: 7–11.

[30] Combosch DJ, Vollmer SV. Population genetics of an ecosystem-defining reef coral *Pocillopora damicornis* in the Tropical Eastern Pacific. PloS One. 2011; 6: e21200. 21857900 10.1371/journal.pone.0021200 PMC3153452

[31] Veron JE, DeVantier LM, Turak E, Green AL, Kininmonth S, Stafford-Smith M, et al. The coral triangle. Coral reefs: an ecosystem in transition. 2011; 47–55.

[32] Bowen BW, Rocha LA, Toonen RJ, Karl SA, ToBo Laboratory. The origins of tropical marine biodiversity. Trends in Ecology & Evolution. 2013; 28: 359–366. 23453048 10.1016/j.tree.2013.01.018

[33] Evans SM, McKenna C, Simpson SD, Tournois J, Genner MJ. Patterns of species range evolution in Indo-Pacific reef assemblages reveal the Coral Triangle as a net source of transoceanic diversity. Biology Letters. 2016; 12: 1–4. doi: 10.1098/rsbl.2016.0090 27330168 PMC4938039

[34] Cacciapaglia C, van Woesik R. Reef‐coral refugia in a rapidly changing ocean. Global Change Biology. 2015; 21: 2272–2282. doi: 10.1111/gcb.12851 25646684

[35] Cinner JE, Huchery C, MacNeil MA, Graham NAJ, McClanahan TR, Maina J, et al. Bright spots among the world’s coral reefs. Nature. 2006; 535: 416–419. 10.1038/nature18607 27309809

[36] Sully S, Hodgson G, van Woesik R. Present and future bright and dark spots for coral reefs through climate change. Global Change Biology. 2022; 28: 4509–4522. doi: 10.1111/gcb.16083 35106864 PMC9303460

[37] Schulman AH. Molecular markers to assess genetic diversity. Euphytica/ Netherlands Journal of Plant Breeding. 2007; 158: 313–321.

[38] Shearer TL, van Oppen MJH, Romano SL, Wörheide G. Slow mitochondrial DNA sequence evolution in the Anthozoa (Cnidaria). Molecular Ecology. 2002; 11: 2475–2487. doi: 10.1046/j.1365-294x.2002.01652.x 12453233

[39] van Oppen MJ, Willis BL, Vugt HW, Miller DJ. Examination of species boundaries in the Acropora cervicornis group (Scleractinia, cnidaria) using nuclear DNA sequence analyses. Molecular Ecology. 2000; 9: 1363–1373. doi: 10.1046/j.1365-294x.2000.01010.x 10972775

[40] NCBI. National Center for Biotechnology Information (NCBI) [cited 01 February 2024] [Internet]. Bethesda (MD): National Library of Medicine (US), National Center for Biotechnology Information; 1988–2024, March 17. Available from: https://www.ncbi.nlm.nih.gov/

[41] Earth Google. Version 10.64.0.3. 2023. https://earth.google.com/

[42] Prime Geneious. Version v.2023.2. 2023. https://www.geneious.com

[43] R Core Team. R: A Language and Environment for Statistical Computing. R Foundation for Statistical Computing, Vienna, Austria. 2023.

[44] Paradis E. pegas: an R package for population genetics with an integrated–modular approach. Bioinformatics. 2010; 26: 419–420. doi: 10.1093/bioinformatics/btp696 20080509

[45] UNEP-WCMC WorldFish Centre, WRI TNC. Global distribution of warm-water coral reefs, compiled from multiple sources including the Millennium Coral Reef Mapping Project. Version 4.1. Includes contributions from IMaRS-USF and IRD (2005), IMaRS-USF (2005) and Spalding et al. (2001). Cambridge (UK): UN Environment World Conservation Monitoring Centre. 2021. Available from: 10.34892/t2wk-5t34

[46] Hijmans RJ. raster: Geographic Data Analysis and Modeling. R package version 3.6–26. 2023.

[47] LeDell E, Gill N, Aiello S, Fu A, Candel A, Click C, et al. h2o: R Interface for the “H2O” Scalable Machine Learning Platform. R Package Version 3.36.0.4. 2022.

[48] Candel A, LeDell E. Deep learning with H2O. Mountain View, California: H2O. ai Inc; 2022.

[49] Molnar C, Bischl B, Casalicchio G. iml: An R package for Interpretable Machine Learning. Journal of Open-Source Software. 2018; 3: 786.

[50] Fisher A, Rudin C, Dominici F. All Models are Wrong, but Many are Useful: Learning a Variable’s Importance by Studying an Entire Class of Prediction Models Simultaneously. Journal of Machine Learning Research 20. 2019; 1–81. doi: 10.1080/01621459.1963.10500830 34335110 PMC8323609

[51] Posada D. jModelTest: phylogenetic model averaging. Molecular biology and evolution. 2008; 25(7): 1253–1256. doi: 10.1093/molbev/msn083 18397919

[52] Yetsko K, Ross M, Bellantuono A, Merselis D, Rodriquez-Lanetty M, Gilg MR. Genetic differences in thermal tolerance among colonies of threatened coral *Acropora cervicornis*: potential for adaptation to increasing temperature. Marine Ecology Progress Series. 2020; 646: 45–68.

[53] Parkinson JE, Banaszak AT, Altman NS, LaJeunesse TC, Baums IB. Intraspecific diversity among partners drives functional variation in coral symbioses. Scientific reports. 2015; 5: 15667. doi: 10.1038/srep15667 26497873 PMC4620489

[54] Gillette P. Intraspecific genetic variability in temperature tolerance in the coral Pocillopora damicornis: Effects on growth, photosynthesis, and survival. Doctoral Dissertation. University of Miami. 2012, pp 88.

[55] Thomas L, Şahin D, Adam AS, Grimaldi CM, Ryan NM, Duffy SL, et al. Resilience to periodic disturbances and the long-term genetic stability in *Acropora* coral. Communications Biology. 2024; 7: 410. 38575730 10.1038/s42003-024-06100-0 PMC10995172

[56] Ayre DJ, Hughes TP. Climate change, genotypic diversity and gene flow in reef‐building corals. Ecology Letters. 2000; 7: 273–278.

[57] Rougerie F, Rancher J. The Polynesian South Ocean: Features and Circulation. Marine Pollution Bulletin 1994; 29: 14–25.

[58] Johnston EC, Wyatt ASJ, Leichter JJ, Burgess SC. Niche differences in co-occurring cryptic coral species (Pocillopora spp.). Coral Reefs. 2022; 41: 767–778.

[59] Huang D, Roy K. The future of evolutionary diversity in reef corals. Phil. Trans. R. Soc. 2015; B37020140010. doi: 10.1098/rstb.2014.0010 25561671 PMC4290424

[60] Ekman S. Zoogeography of the Sea. Translated from Swedish by Elizabeth Palmer. London: Sidgwick and Jackson. New York: Macmillan; 1953, pp 417.

[61] Palumbi SR. Molecular biogeography of the Pacific. Coral Reefs. 1997; 16: S47–S52.

[62] Woodland DJ. Zoogeography of the Siganidae (Pisces): An Interpretation of Distribution and Richness Patterns. Bulletin of Marine Science. 1983; 33: 713–717.

[63] Bellwood DR, Meyer CP. Searching for heat in a marine biodiversity hotspot. Journal of Biogeography. 2009; 36: 569–576.

[64] Ladd HS. Origin of the Pacific Island molluscan fauna. American Journal of Science. 1960; 256: 137–150.

[65] Chaudhary A, Kastner T. Land use biodiversity impacts embodied in international food trade. Global Environmental Change: Human and Policy Dimensions. 2016; 38: 195–204.

[66] Yasuhara M, Wei C-L, Kucera M, Costello MJ, Tittensor DP, Kiessling W, et al. Past and future decline of tropical pelagic biodiversity. Proceedings of the National Academy of Sciences of the United States of America. 2020; 117: 12891–12896. doi: 10.1073/pnas.1916923117 32457146 PMC7293716

[67] Gélin P, Pirog A, Fauvelot C, Magalon H. High genetic differentiation and low connectivity in the coral *Pocillopora damicornis* type β at different spatial scales in the Southwestern Indian Ocean and the Tropical Southwestern Pacific. Marine Biology. 2018; 165: 167.

[68] Palumbi SR, Grabowsky G, Duda T, Geyer L, Tachino N. Speciation and population genetic structure in tropical Pacific sea urchins. Evolution; International Journal of Organic Evolution. 1997; 51: 1506–1517. 10.1111/j.1558-5646.1997.tb01474.x 28568622

[69] Baums IB, Boulay JN, Polato NR, Hellberg ME. No gene flow across the Eastern Pacific Barrier in the reef-building coral Porites lobata. Molecular Ecology. 2012; 21: 5418–5433. doi: 10.1111/j.1365-294X.2012.05733.x 22943626

[70] Thompson DM, van Woesik R. Corals escape bleaching in regions that recently and historically experienced frequent thermal stress. Proceedings of the Royal Society B. 2009; 276: 2893–2901. doi: 10.1098/rspb.2009.0591 19474044 PMC2817205

