# Supplementary material for: Geographical patterns of intraspecific genetic diversity reflect the adaptive potential of the coral Pocillopora damicornis species complex
Source: PLoS One. 2025 Jan 22;20(1):e0316380. doi: 10.1371/journal.pone.0316380 (PMC11753671; doi:10.1371/journal.pone.0316380)
Supplement: S1 File — (PDF) [file pone.0316380.s001.pdf]

Supplementary document for

Geographical patterns of intraspecific genetic diversity reflect the adaptive potential of the coral *Pocillopora damicornis*

M. Carr, C. Kratochwill, T. Daly-Engel, T. Crombie, R. van Woesik

Quality control for genetic sequences

After each of the 8 fasta files was created, they were converted into R as.DNAbin() or read in as DNAbin objects using the package *pegas* for analysis (Paradis, 2010). Each DNAbin file had sequences renamed according to their site location, and a geneid file was created with groupings by the site. Next, the nucleotide diversity was calculated using nuc.dif() function from "pegas". Results were copied and pasted into a comma-separated-variable (CSV) file and uploaded into R.

The raw downloaded sequences were cleaned using the following seven methods in which the sequence ends were: (i) trimmed, (ii) trimmed and indels removed, (iii) trimmed and cleaned, (iv) cleaned and one gap left for indels, (v) trimmed, cleaned, and all indels removed, (vi) trimmed, cleaned, indels removed, and transversions cleaned, and (vii) trimmed, cleaned, indels removed, and point mutation cleaned (all data are available at https://github.com/InstituteForGlobalEcology/Pocillopora).

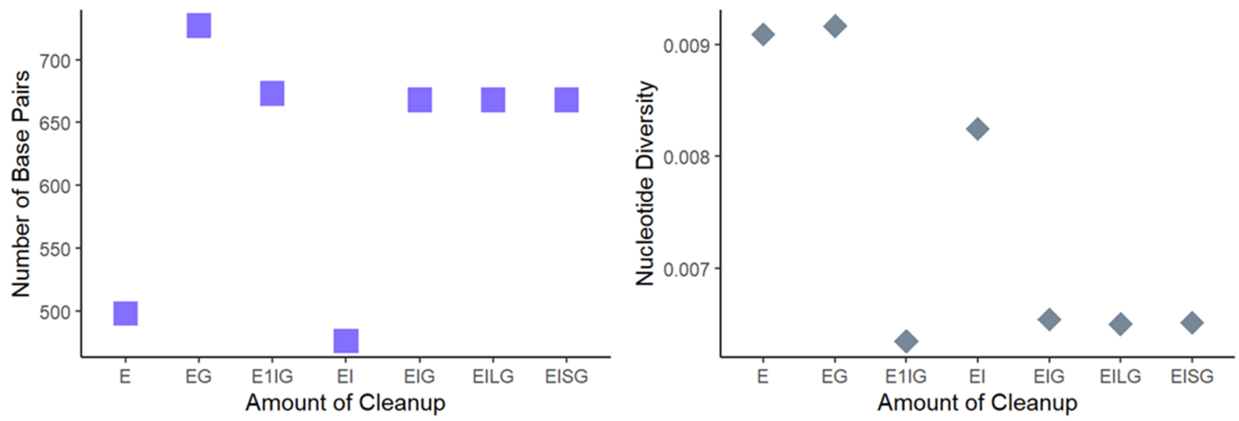

Figure S1. Response to clean up for the number of base pairs and nucleotide diversity for 44 *Pocillopora damicornis* sampling sites in the Pacific and Indian Oceans from 2005 to 2020. Genbank raw data were: (i) trimmed (E), (ii) trimmed and indels removed (EI), (iii) trimmed and cleaned (EG), (iv) cleaned and one gap left for indels (E1IG), (v) trimmed, cleaned, and all indels removed (EIG), (vi) trimmed, cleaned, indels removed, and transversions cleaned (EILG), and (vii) trimmed, cleaned, indels removed, and point mutation cleaned (EISG).

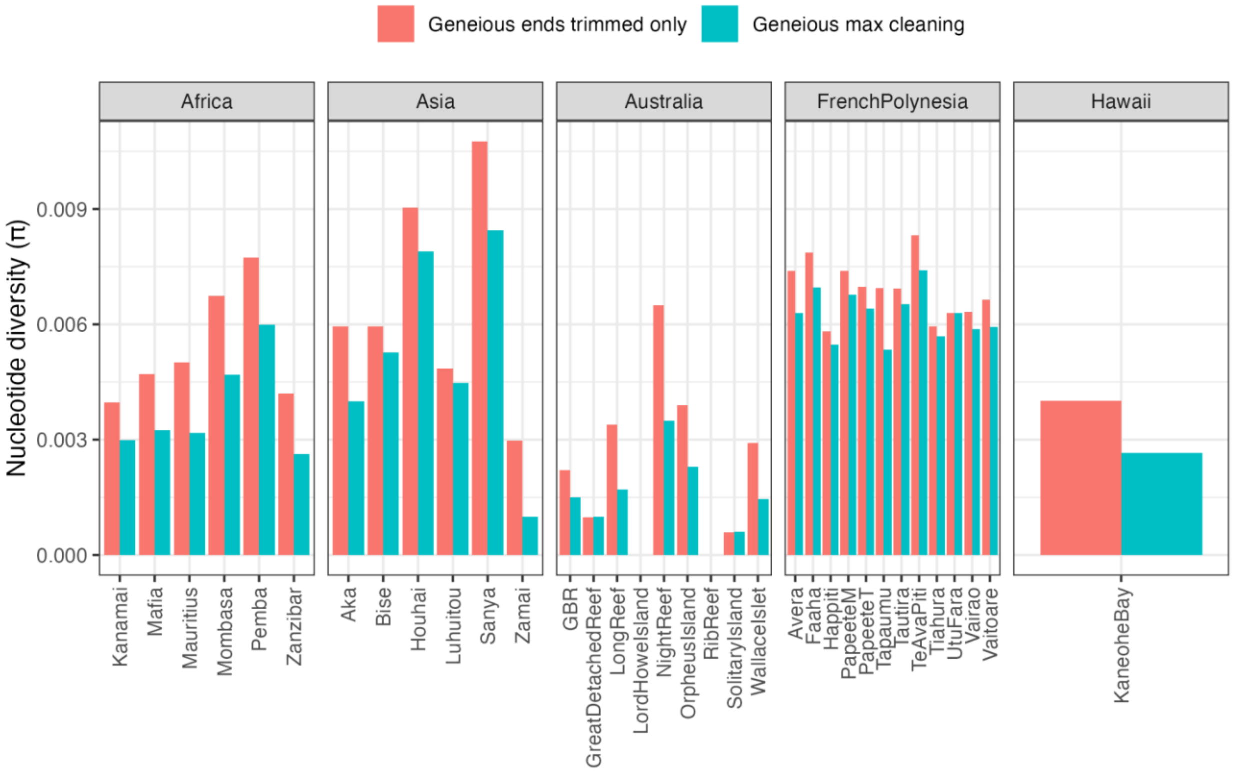

Figure S2. Comparative nucleotide diversity of the coral *Pocillopora damicornis* among 34 sites (with ≥ 3 samples) in the Pacific and Indian Oceans from 2005 to 2020. The data with the "Geneious ends trimmed only" are orange and the "Geneious max cleaning" (i.e., with the ends trimmed, cleaned, indels removed, and point mutation cleaned) are green.

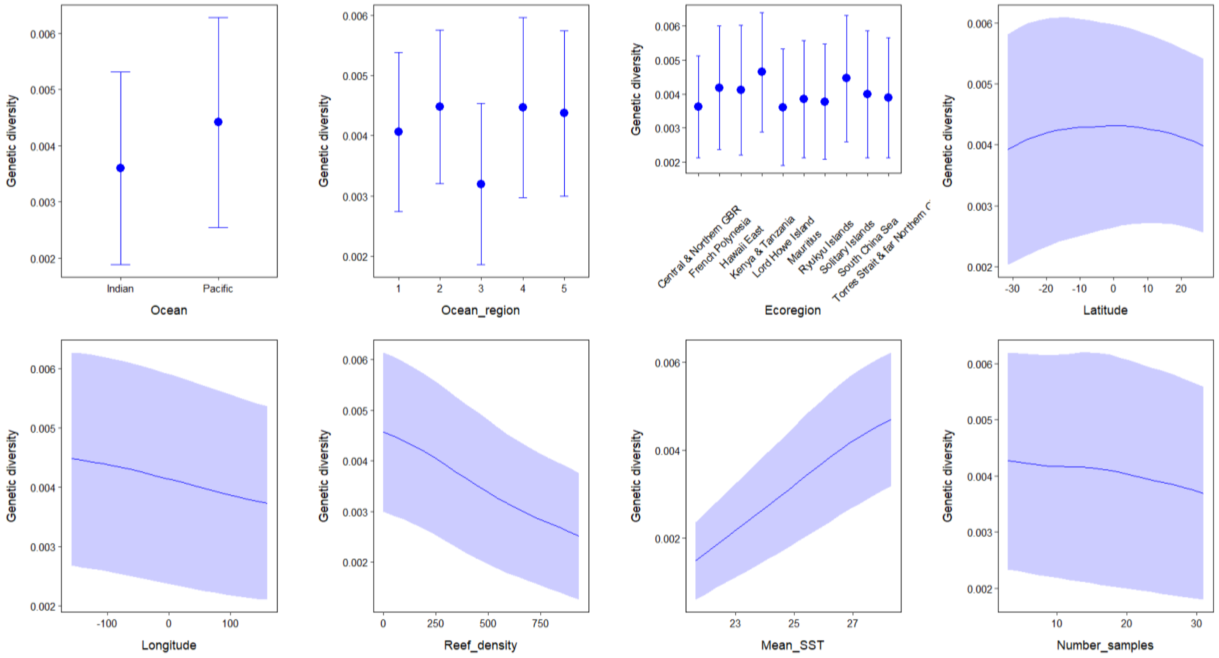

Figure S3. Variable importance plot using results from the deep-learning neural-network model showing the relative influence of eight predictor variables on the nucleotide (i.e., genetic) diversity of the coral *Pocillopora damicornis* at 44 sampling sites in the Pacific and Indian Oceans from 2005 to 2020. The data used here had the ends trimmed, cleaned, indels removed, and point mutation cleaned (compare this plot to Figure 4 in the main paper). Where Reef density is the number of reef centroids within a 500 km (250 km radius) of a given study site, num = number, and SST is the sea-surface temperature (ºC).

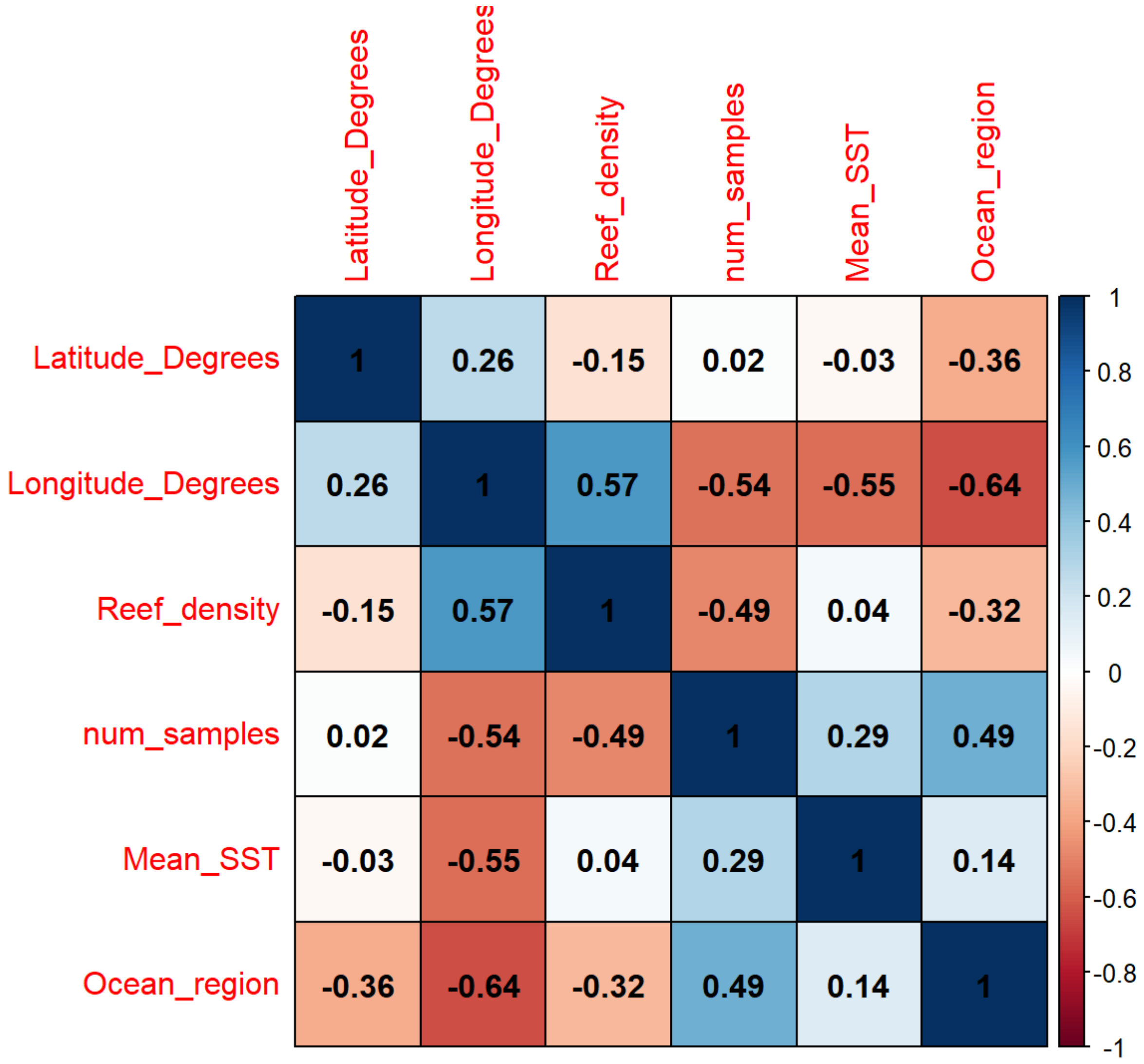

Figure S4. Correlation plot for the six continuous predictor variables used in Deep Learning Neural Network for 44 *Pocillopora damicornis* sampling sites in the Pacific and Indian Oceans from 2005 to 2020. The tone of the color resembles the strength of the correlation between variables with the darker tones representing a stronger correlation than the lighter tones. Where Reef density is the number of reef centroids within 500 km (i.e., 250 km radius) of a given study site, num = number, and SST is the sea-surface temperature (ºC).

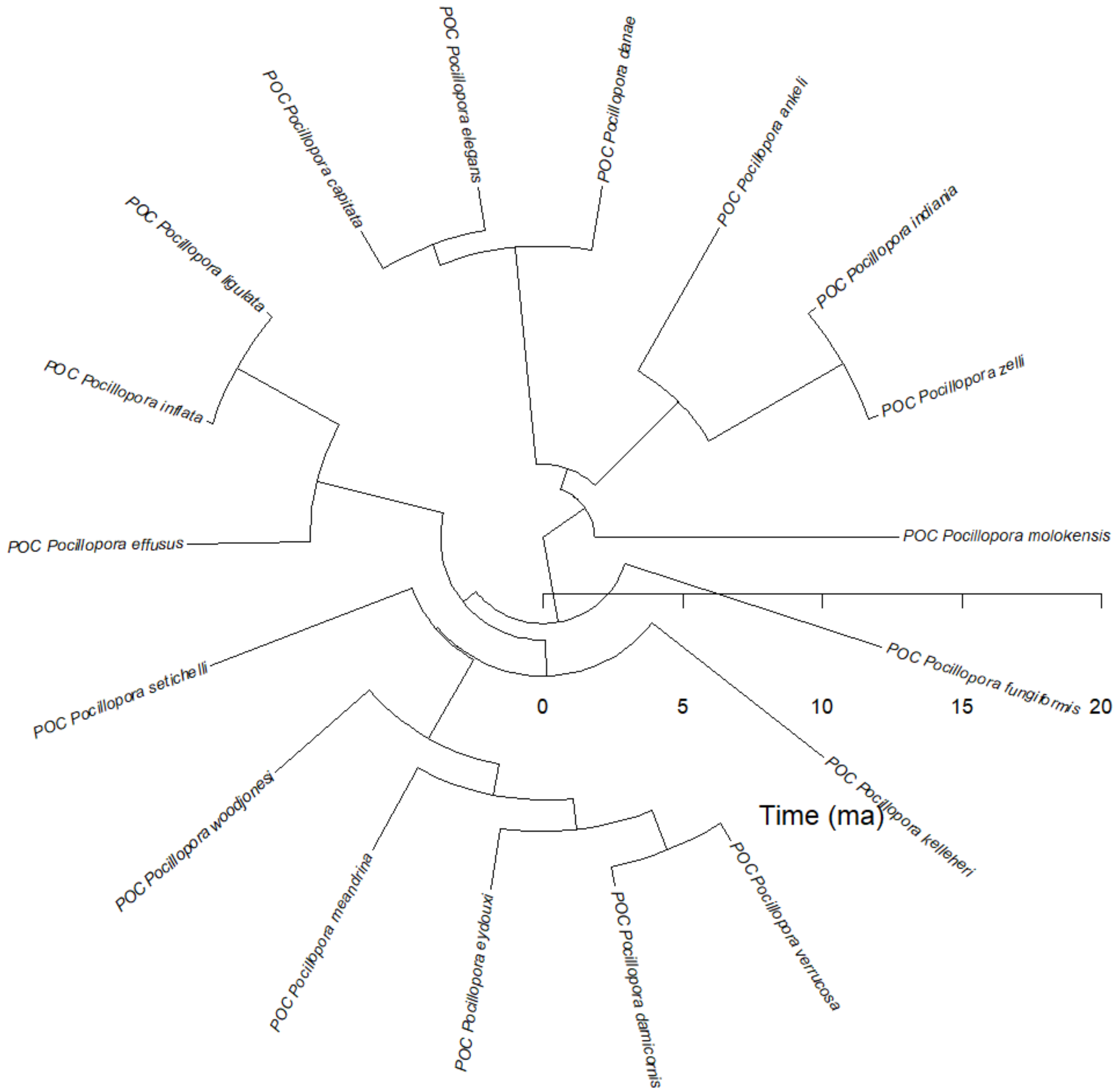

Figure S5. *Pocillopora* phylogenetic tree created using data from Huang & Roy (2015) The future of evolutionary diversity in reef corals, Phil. Trans. R. Soc. B37020140010. Data accessed via Dryad http://dx.doi.org/10.5061/dryad.178n3 (September 6, 2024).
