# Supplementary figures and images for: Geographical patterns of intraspecific genetic diversity reflect the adaptive potential of the coral Pocillopora damicornis species complex
Source: PLoS One. 2025 Jan 22;20(1):e0316380. doi: 10.1371/journal.pone.0316380 (PMC11753671; doi:10.1371/journal.pone.0316380)

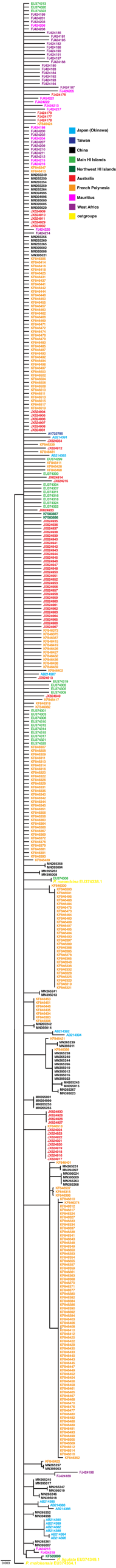

Supplement: S2 File — (PDF) [file pone.0316380.s002.pdf]
